# Dysfunctional Metacognitive Beliefs in Body Dysmorphic Disorder

**DOI:** 10.5539/gjhs.v8n3p10

**Published:** 2015-06-20

**Authors:** Zahra Zeinodini, Sahar Sedighi, Mandana Baghertork Rahimi, Simasadat Noorbakhsh, Sepideh Rajezi Esfahani

**Affiliations:** 1 Behavioral Sciences Research Center of Shahid Beheshti University of Medical Sciences, Tehran, Iran

**Keywords:** body dysmorphic disorder, cognitive therapy, cognition, adolescent, metacognitive beliefs

## Abstract

The present study aims to examine the correlation of body dysmorphic disorder, with metacognitive subscales, metaworry and thought-fusion. The study was conducted in a correlation framework. Sample included 155 high school students in Isfahan, Iran in 2013-2014, gathered through convenience sampling. To gather data about BDD, Yale-Brown Obsessive Compulsive Scale Modified for BDD was applied. Then, Meta Cognitive Questionnaire, Metaworry Questionnaire, and Thought-Fusion Inventory were used to assess metacognitive subscales, metaworry and thought-fusion. Data obtained from this study were analyzed using Pearson correlation and multiple regressions in SPSS 18. Result indicated YBOCS-BDD scores had a significant correlation with scores from MCQ (P<0.05), MWG (P<0.05), and TFI (P<0.05). Also, multiple regressions were run to predict YBOCS from TFI, MWQ, and MCQ-30. These variables significantly predicted YBOCS [F (3,151) =32.393, R^2^=0.57]. Findings indicated that body dysmorphic disorder was significantly related to metacognitive subscales, metaworry, and thought fusion in high school students in Isfahan, which is in line with previous studies. A deeper understanding of these processes can broaden theory and treatment of BDD, thereby improve the lives of sufferers and potentially protect others from developing this devastating disorder.

## 1. Introduction

Known as dysmorphophobia, Body Dysmorphic Disorder (BDD) is a mental health problem which is chronic and disabling, known by a preoccupation with an imagined deformity in appearance. BDD is characterized by problematic disruptions in self-esteem, time-consuming repetitive actions and avoidance (e.g., of social interactions, mirrors, lights), which typically starts in adolescence and has a mean age of onset at 16 years and mode of 13 years (Phillips, Menard, Fay, & Weisberg, 2005). The overall point prevalence in general population, equal in both genders, is 0.7-2.4% (Gupta, Huynh, & Ginsburg, 2013), moreover its rates were higher in patients with social phobia and obsessive-compulsive disorder (Wells, 2010).

One route to a deeper understanding of the processes underlying the psychopathology of BDD is examining the cognitive mechanisms involved in the knowledge, interpretation, and regulation of thinking itself. These mechanisms comprise the domain of ‘metacognition’ or thinking about thinking (Wells, 2011). The dominant theory regarding the role of metacognition in psychopathology is self-regulatory executive function (S-REF) theory (Wells, 2011), which identifies two basic components of metacognition: knowledge and regulation. According to the theory, metacognitive knowledge consists of the beliefs an individual holds about the course and consequences of cognitive enterprises. This knowledge may be accurate or inaccurate, explicit or implicit, and can be triggered unintentionally by retrieval cues (Wells, 2013). Metacognitive regulation involves executive functions such as planning, resource allocation, monitoring, and correcting of cognitive events. In S-REF theory, psychological disorder is associated with dysfunction of this system, in a way that the regulation and knowledge processes become maladaptive (Wells, 2010, 2011).

One of the fundamental concepts of metacognitive model is thought-fusion in which metacognitive beliefs eliminate the borders between thought, incidents and acts (Gladstone et al., 2005). On the other hand, worry as a key part of distress has been described as a chain of thoughts and images and relatively automatic thoughts related to possible threatening outcomes and their potential consequences (Cooper & Osman, 2007). Worry is widespread both in people with disorders or those without, which varies in degree existing among people rather than the quality of its existence. Uncontrollability of worry might cause drastic interference and distress in daily life; in addition, metaworries are considered as problematic components of metacognition. Also, worry can become the focus of anxious apprehension. Studies by Cooper and Osman (Cooper & Osman, 2007) showed that people with BDD had completely different metacognitive thought that they were unattractive, ugly, inferior, and worthless. Metacognitive control strategies, defined as those strategies used to control the cognitive system, are not only likely to strengthen or suppress mental strategies but to increase monitoring processes as well.

Cross-sectional and directional relationships between maladaptive metacognition and a wide range of psychological dysfunctions have been extensively demonstrated. These include depression (Wells, 2011), GAD (Wells, 2010), PTSD (Wells, Walton, Lovell, & Proctor, 2014), obsessive-compulsive disorder (Barahmand & Shahbazi, 2013), eating disorders (McDermott & Rushford, 2011), and psychosis (Hutton, Morrison, Wardle, & Wells, 2014). If S-REF theory is correct in identifying dysfunctional metacognition as a generic vulnerability factor underlying psychopathology, this should be also held true for BDD. If metacognitive factors were proved important in BDD, this would invite the application of MCT to its treatment.

The present research aims to investigate the relationships between dysfunctional metacognition and body dysmorphic disorder.

## 2. Method

The present research, which was conducted within a correlation framework, examined the relationships between dysfunctional metacognitive variables and body dysmorphic disorder. Population included all male and female students who studied in high school in 2010-2011 in Isfahan, Iran.

### 2.1 Participants

Current study is a correlation study. To determine the sample size, the Cochran's sample size formula for correlation study was used:


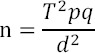


in which T=1.96, p=0.5, q=0.5, and d=0.11. The minimum sample size needed to obtain statistically valid results was 79, and we reduplicated samples to obtain higher validity.

Research sample included 155 adolescent students aged between 12 and 17 years who were selected through convenient sampling from three high schools, which are governmental educational center in Isfahan (2013-2014). Individuals above the age of 17 years or below the age of 12 years were not considered, as their prevalence in the BDD population is small to allow meaningful statistical analysis. Students who were too medically or psychologically too compromised to give informed consent were not considered. Since less information is available about rates of body dysmorphic disorder in addition to the fact that such patients are more linked to dermatologists and plastic surgeons rather than psychiatrists or psychologists (Gupta et al., 2013), the current research focused mainly on students in high school. Inclusion and Exclusion criteria are presented in Table 1. According to exclusion criteria, 28 participants excluded.

**Table 1:** Inclusion and Exclusion criteria

Inclusion Criteria	Exclusion Criteria
Having an age range of 12-17	Receiving pharmacotherapy or psychotherapy
Satisfaction to participate	Having psychosis or schizophrenia
Written informed consent form	Having personality disorders
	Not willingness to participate

### 2.2 Procedure

Participants were approached by the researchers, who explained the project, providing plain language statements (PLS), and answered queries. Students were assured that their decision regarding participation would not affect anything at school. Those who agreed to participate signed a consent form and were given a questionnaire set to return later in a sealed envelope to the researchers. To protect anonymity, the researchers did not view questionnaires completed by their own acquaintances. All participants received an ID enabling them to withdraw their information should they change their mind about participating. Completion of the questionnaire set required approximately 40 minutes. All of the tests were conducted by two school psychologists in an office one by one.

### 2.3 Measures

All participants were assessed by Yale Brown Obsessive Compulsive Scale Modified for BDD to find out whether they have BDD. Thought-Fusion Instrument and Short Form of the Metacognition Questionnaire have also been used to investigate their dysfunctional metacognition.

Yale Brown Obsessive Compulsive Scale Modified for BDD: YBOCS-BDD is a 12-item questionnaire, rated by a clinician. It has questions on preoccupations (5), compulsive behaviors (5), insight (1), and avoidance (1). Mostly, it was developed as a measure of severity of BDD symptoms. Each item is scored in 5-point Likert's scale from 0 (totally agree) to 4 (totally disagree). Philips et al (Phillips, Hollander, Rasmussen, & Aronowitz, 1997) have confirmed the reliability of YBOCS-BDD through interrater and test-retest. YBOCS-BDD was reported to have positive correlation (r=0.51) with Global Assessment of Functioning in DSM. Rabiei et al. (Rabiei, Khormdel, Kalantari, & Molavi, 2010) examined the factor structure, validity and reliability of the Modified Yale-Brown Obsessive-Compulsive Scale in a sample of Iranian students. They found that YBOCS-BDD had satisfactory reliability and validity in the sample of Iranian students, and could therefore be used for diagnostic and therapeutic purposes (Rabiei et al., 2010).

Thought-Fusion Instrument (TFI): TFI consists of 14 items rated on a 0 to 100 scale which assess metacognitive beliefs about the meaning, importance, and peril of intrusive thoughts. It was designed to measure the three types of thought fusions: Thought-Action Fusion, Thought-Event Fusion and Thought-Object Fusion. Gwilliam et al (Gwilliam, Wells, & Cartwright-Hatton, 2004) obtained acceptable reliability and preliminary evidence supports its convergent and discriminate validity. Also, other studies have showed the correlation from 0.4 to 0.7 between TFI and metacognitive beliefs instrument and thought action fusion (Rachman, Thordarson, Shafran, & Woody, 1995). Khoramdel et al have reported satisfactory reliability and validity in Iranian students population which can be used for diagnosis and treatment (Khoramdel, Rabiee, Molavi, & Neshatdoost, 2010).

A Short Form of the Metacognition Questionnaire (MCQ-30): it has 30 multiple choice items which are in ranges of totally disagree (1 point), partially agree (2 points), mildly agree (3 points) and totally agree (4 points). MCQ measures five components including 1) cognitive confidence, 2) positive beliefs about worry, 3) cognitive self-consciousness, 4) negative beliefs about thoughts and danger which are out of control, and 5) beliefs about demand to control thoughts (Wells & Cartwright-Hatton, 2004). The Cronbach's alpha coefficient for all questions was reported in a range of 0.72 to 0.93, and the test-retest reliability coefficient of the Short From of the Metacognition Questionnaire was reported 0.73 (Wells & Cartwright-Hatton, 2004). It also demonstrated acceptable psychometric properties in Iranian population (Shirinzadeh, Goudarzi, Ghanizadeh, & Taghavi, 2008).

Metaworry Questionnaire (MWQ): The questionnaire has 7 items which measure worry and metacognition. There are two scales for each item. One to assess the frequency of meta-worry which is a Likert's scale ranging from 1 to 4 with each point labeled as follows: Never; sometimes; often; almost always. The other is used to rate the belief in each meta-worry at its time of occurrence and ranges from 0 to 100 with anchor points labeled at each extreme as follows: I do not believe this thought at all, and I am completely convinced this thought is true (Wells, 2005). It has very good internal reliability, and the scales correlated meaningfully with existing measures. Cronbach's alpha coefficients of the MWQ were 0.88 for the frequency scale and 0.95 for the belief scale. The meritorious and marvelous criteria were respectively satisfying the MWQ frequency subscale (0.87), and the belief subscale (0.93). The variables were inter-correlated because Bartlett's test statistic (p<.0005) was highly significant for both the frequency and belief variables. MWQ scales were positively correlated with AnTI metaworry subscale, negative beliefs about worry measured with the MCQ social worry, health worry, and positive worry beliefs. Males and females were found not to differ significantly on each of the subscales (Wells, 2005).

### 2.4 Ethical Aspects

The Study was approved by the Ethical Review Board of the Behavioral Sciences Research Center of Shahid Beheshti University of Medical Sciences, Tehran, Iran (1393-1-102-1356-1, 10. Feb. 2013). Informed consent forms were obtained from all participants. Before starting the study, in a formal session in school, the participants and their parents were provided with a general overview of the goals and aspects of the study. They were also informed that they were participating voluntarily, and that they could leave the study at any time without any negative consequences. The results were used anonymously and all of the data were kept secret in this study.

### 2.5 Statistical Analyses

Analyses were performed using the Statistical Package for Social Sciences (SPSS) version 18. Both descriptive and inferential statistics had been used to find the possible correlation between BDD and dysfunctional metacognition. Data obtained from this study were analyzed using Pearson correlation and multiple regressions.

## 3. Results

In Table 2, demographic information of participants is presented.

**Table 2:** Demographic characteristics of participants

Variables	Levels of variables	frequency	percentage
Gender	Male	77	49.5
	Female	78	50.5
History of hospitalization	Yes	17	10.96
	No	138	89.4
History of academic failure	Yes	12	7.74
	No	143	92.26
Father's education	Diploma	36	23.22
	undergrad	93	60
	Post grad	26	16.78
Mother's education	Diploma	89	57.41
	undergrad	46	29.67
	Post grad	20	12.92

Table 3 shows the Pearson correlation matrix of YBOCS-BDD with MCQ-30, MWQ, and TFI.

**Table 3:** Correlation matrix among metacognitive components, thought confusion, metaworry and body dysmorphic disorder

	YBOCS-BDD	MCQ	MWQ	TFI
YBOCS-BDD	1			
MCQ	0.39*	1		
MWQ	0.42*	0.33	1	
TFI	0.35*	0.32	0.44	1

* p< 0.05.

As shown in Table 3, the students’ scores in all dysfunctional metacognition components are related to the body dysmorphic disorder significantly. In other words, YBOCS-BDD scores demonstrated a significant relationship with MCQ (r=0.39, P<0.05), MWQ (r=0.42, P<0.05), and TFI (r=0.35, P<0.05).

Tables 4 and 5 present the results of multiple regressions to predict YBOCS from TFI, MWQ and MCQ-30 scores.

**Table 4:** The results of multiple regressions for TFI, MWQ, and MCQ-30 in high school students

Model		Sum of Squares	df	Mean Square	F	P value
1	Regression	270.032	3	90.011	32.393	.000^b^
	Residual	2282.608	151	108.696		
	Total	2552.640	154			

a. Dependent Variable: YBOCS;

b Predictors: (Constant), TFI, MWQ, MCQ-30.

**Table 5:** The results of multiple regressions for TFI, MWQ, and MCQ-30 in high school students

Model		Unstandardized Coefficients		Standardized Coefficients	t	P value	95.0% Confidence Interval for B	
	
		B	Std. Error	Beta			U	L
1	(Constant)	35.439	13.334		2.658	.000	7.710	63.168
	TFI	-.411	.478	-.178	-.859	.010	-1.406	.584
	MWQ	.248	.468	.110	.530	.047	-.726	1.222
	MCQ-30	-.438	.417	-.220	-1.050	.006	-1.304	.429

a. Dependent Variable: YBOCS; p<.05.

As shown in Table 5, a multiple regression was run to predict YBOCS from TFI, MWQ, and MCQ-30. These variables statistically significantly predicted YBOCS [F (3,151) =32.393, p< 0.0005, R^2^=0.577]. All variables added statistically significantly to the prediction (p<0.05).

## 4. Conclusions

The present research aimed to examine the relationship between dysfunctional metacognition with body dysmorphic disorder (BDD). Findings show that BDD has a significant positive relationship with thought fusion, metaworry, and dysfunctional metacognition, which is consistent with previous studies (Cooper & Osman, 2007; Fairfax, 2008; Holmes, Arntz, & Smucker, 2007; McDermott & Rushford, 2011; Toh, Rossell, & Castle, 2009; Veale et al., 2014).

BDD patients are engaged in dysfunctional metacognition about their body appearance (Cooper & Osman, 2007). The metacognition components of body dysmorphic disorder include the strategies for metacognitive controlling such as suppressing thoughts about being ugly, worries about dysmorphic, rumination avoiding, reassurance seeking or excessive grooming (Cooper & Osman, 2007). According to Veale (Veale et al., 2014), in BDDs, metacognition is a noticeable issue in information processing, and it possibly aids maintenance of symptoms. Considering the relationships between metacognitive components and body dysmorphic disorder, it is worth of notice that the scores of components could successfully predict the disorder (Arbel, Koren, Klein, & Latzer, 2013; McDermott & Rushford, 2011; Wells, 2013).

The demonstrated importance of dysfunctional metacognition in BDD illuminates a possible mechanism for the inefficacy of CBT in its treatment (Toh et al., 2009). By focusing exclusively on the content of thoughts, CBT neglects the crucial role played by cognitive processes underlying these thoughts. S-REF theory contends that these metacognitions generate the problematic thought content challenged in CBT; therefore, merely modifying that content without addressing its underlying source is unlikely to prove effective in the long term. To overcome this limitation, Wells (Wells, 2011) developed metacognitive therapy, which aims to decrease dysfunctional metacognitive beliefs and strategies and teach the individual new ways of consciously experiencing cognitive events.

Given its demonstrated effectiveness in several psychological disorders (Wells, 2011), the present results suggest that metacognitive therapy may hold great therapeutic potential for BDD. The positive correlation between metacognitive beliefs and scores of body dysmorphic disorder in high school student could have implication for practice. According to wells (Wells, 2010), psychiatric disorders are caused by metacognitive thoughts which can be controlled or changed. Thus, recognizing thoughts of BDD patients can be the initial step to design and practice metacognitive therapeutic method for those patients.

The current study is one of the first to demonstrate that metacognitive dysfunction may play a key role in BDD. This study had some limitations including the fact that the groups were not homogeneous, the age and education level of applicants were not various enough and interview, due to expensive implementation costs, was not employed as an instrument in selecting applicants. It is suggested that a parallel study should be conducted for the applicants with different age and education level in homogenous groups. The findings should be interpreted in the context of certain methodological limitations which should be addressed in future research. First, the cross-sectional design precludes causal conclusions. Longitudinal studies of the relationships between metacognition and BDD symptoms, as well as experimental manipulations of these variables, and studies on clinical and nonclinical populations may provide evidence of causality. Second, the relatively small sample limits generalizability; therefore a large, multi-site study should be established to determine if these findings are replicable. To substantiate the specificity of the results to BDD, a control group from patients with body dysmorphic disorder should be included. Furthermore, as self-report measures are intrinsically prone to idiosyncratic interpretation and demand characteristics, response authenticity should be strengthened by use of implicit measures and interviews. Finally, future studies should consider depression, anxiety and other psychological disorders as confounding variables.

In conclusion, findings indicated that body dysmorphic disorder was significantly related to metacognitive subscales, metaworry, and thought fusion in high school students in Isfahan, which is in line with previous studies.
